# Circulating NET-associated parameters across chronic inflammatory skin diseases: a comparative study

**DOI:** 10.3389/fmed.2026.1918335

**Published:** 2026-10-23

**Authors:** Joanna Czerwińska, Agnieszka Markiewicz, Alicja Frączek, Maja Kleniewska, Marta Kasprowicz-Furmańczyk, Agnieszka Owczarczyk-Saczonek

**Affiliations:** Department of Dermatology, Sexually Transmitted Diseases and Clinical Immunology, School of Medicine, Collegium Medicum, University of Warmia and Mazury in Olsztyn, Olsztyn, Poland

**Keywords:** acne inversa, NETosis (Neutrophil extracellular traps), psoriasis, pyoderma gangrenosum, vitiligo

## Abstract

**Background:**

Neutrophil extracellular traps (NETs) are important components of innate immunity and may contribute to immune dysregulation in chronic inflammatory skin diseases. However, circulating NET-associated profiles across dermatoses with distinct immunopathogenic characteristics remain incompletely characterized. This study aimed to compare circulating NET-associated parameters in hidradenitis suppurativa (HS), pyoderma gangrenosum (PG), vitiligo (VIT), and psoriasis (PsO).

**Methods:**

Serum MPO–DNA complexes, representing circulating NET-associated remnants, peptidylarginine deiminase 4 (PAD-4), a NET formation-associated marker, and DNase I concentration, a NET-related regulatory marker, were measured using ELISA-based assays. Associations between NET-associated parameters and conventional inflammatory markers, including C-reactive protein (CRP) and absolute neutrophil counts, were also explored. Between-group differences were assessed using the Kruskal–Wallis test followed by *post hoc* multiple comparisons, while correlations were evaluated using Spearman's rank correlation with Benjamini–Hochberg false discovery rate correction. Analyses were exploratory and hypothesis-generating.

**Results:**

Significant between-group differences were observed for MPO–DNA, DNase I, and PAD-4. MPO–DNA levels were significantly higher in moderate-to-severe PsO than in healthy controls, whereas no other pairwise differences reached statistical significance. DNase I concentrations showed a distinct pattern, with higher levels in HS, PG, and VIT than in selected psoriasis groups and/or healthy controls. PAD-4 levels were significantly higher in moderate-to-severe PsO than in VIT, mild PsO, and healthy controls, while PG showed higher PAD-4 levels than VIT. Correlations between NET-associated parameters and conventional inflammatory or clinical measures were generally limited and none remained statistically significant after correction for multiple testing.

**Conclusions:**

Circulating NET-associated parameters differed across chronic inflammatory dermatoses, supporting heterogeneous rather than uniform NET-associated profiles. Moderate-to-severe psoriasis was characterized by higher MPO–DNA and PAD-4 levels relative to healthy controls, whereas HS, PG, and VIT showed comparatively higher DNase I concentrations in selected between-group comparisons. These findings indicate heterogeneity in circulating NET-associated parameters across diseases with distinct immunopathological backgrounds. However, the results should be considered exploratory and hypothesis-generating, as circulating surrogate markers cannot directly establish tissue-level NET formation or degradation. Further studies incorporating orthogonal NET-associated markers, functional assays, and tissue-level analyses are warranted.

## 1 Introduction

Neutrophil extracellular trap (NET) formation (NETosis) is a regulated process in which activated neutrophils decondense chromatin and release extracellular DNA–protein complexes. Canonical NETosis involves peptidylarginine deiminase 4 (PAD-4)-dependent histone citrullination, NADPH oxidase-derived reactive oxygen species (ROS), and disintegration of nuclear and granular membranes, enabling chromatin to associate with granular proteins such as neutrophil elastase (NE), myeloperoxidase (MPO), and antimicrobial peptides, including LL-37, before extracellular release (1–3). Under physiological conditions, NETs serve as an effector mechanism of innate immunity, contributing to pathogen trapping, neutralization, and containment, while also amplifying inflammatory signaling through cytokine and chemokine release (4, 5). Dysregulated NET formation and/or clearance has been implicated in chronic inflammatory and autoimmune diseases. Proposed mechanisms include excessive ROS-driven NET release, altered DNase-mediated degradation leading to NET persistence, and exposure of nuclear antigens that may promote autoantibody formation, including anti-dsDNA, anti-histone, anti-nucleosome, and anti-citrullinated protein antibodies (6–8). Persistent NET accumulation may contribute to inflammation, microvascular dysfunction, and tissue injury. Although NET involvement has been described in inflammatory dermatoses, comparative analyses of circulating NET-associated parameters across diseases with distinct immunopathological backgrounds remain limited. To address this gap, we investigated circulating NET-associated parameters across chronic inflammatory skin diseases with distinct immunopathological backgrounds, including neutrophilic dermatoses (hidradenitis suppurativa [HS] and pyoderma gangrenosum [PG]) and immune-mediated inflammatory diseases (psoriasis [PsO] and vitiligo [VIT]).

HS is characterized by neutrophil-rich dermal inflammation and systemic immune activation and is frequently associated with metabolic and inflammatory comorbidities (9–13). Its pathogenesis involves dysregulated innate and adaptive immune responses, including IL-1β, TNF-α, IL-17, and IL-36 signaling pathways, resulting in persistent tissue inflammation and damage (12–14). PG is a severe neutrophilic dermatosis driven by dysregulated innate immunity and IL-1-mediated inflammation, often associated with systemic inflammatory or autoinflammatory conditions (15, 16). Both HS and PG belong to a spectrum of neutrophil-driven inflammatory disorders characterized by sterile inflammation and sustained innate immune activation. VIT was included as a biologically contrasting chronic inflammatory dermatosis with a distinct immunopathogenic background, rather than as a neutrophilic dermatosis, to explore whether circulating NET-associated parameters vary across inflammatory skin diseases with different immune profiles. Although classically considered a T-cell-mediated disease, increasing evidence suggests a contribution of oxidative stress and innate immune activation, including elevated neutrophil-associated inflammatory markers such as the neutrophil-to-lymphocyte ratio and calprotectin (17–22). PsO is a chronic immune-mediated inflammatory disease in which neutrophils play an important role. NET-associated complexes, particularly LL-37–DNA complexes, have been implicated in dendritic cell activation and downstream type I interferon, IL-23, and IL-17 signaling, contributing to Th1/Th17/Th22 responses and keratinocyte hyperproliferation (23–26).

Collectively, these conditions represent inflammatory dermatoses with distinct immunopathological backgrounds but varying involvement of neutrophil activation, oxidative stress, and innate–adaptive immune crosstalk. We hypothesized that circulating NET-associated parameters would exhibit heterogeneous patterns across these diseases. To explore these differences, we quantified circulating MPO–DNA complexes as NET-associated remnants, PAD-4 as a NET formation-associated marker, and DNase I concentration as a NET-related regulatory parameter. We further explored their associations with conventional inflammatory and clinical parameters. Given that these circulating surrogate markers do not directly measure NET formation or degradation within lesional skin, the analyses were designed as exploratory and hypothesis-generating.

## 2 Results

### 2.1 Peripheral blood inflammatory parameters

Peripheral blood analysis revealed disease-specific differences in systemic inflammatory markers. The highest neutrophil counts and C-reactive protein (CRP) levels were observed in hidradenitis suppurativa (HS) (6.94 ± 0.49 × 10^3^/µL and 9.45 ± 2.45 mg/L, respectively) and pyoderma gangrenosum (PG) (5.90 ± 1.04 × 10^3^/µL and 13.94 ± 3.46 mg/L), with CRP values exceeding the upper reference limit (<5 mg/L) in both groups. Lower values were observed in vitiligo (VIT), mild and moderate-to-severe psoriasis (PsO), and healthy controls.

### 2.2 NET assesment

Next, serum concentrations of NET-associated parameters were analyzed, including MPO–DNA complexes as circulating NET-associated remnants, PAD-4 as a NET formation-associated marker, and DNase I as a NET-related regulatory marker (Figures 1–3, Table 1). In addition, correlations between these parameters and clinical and conventional inflammatory indices were explored.

**Figure 1:**
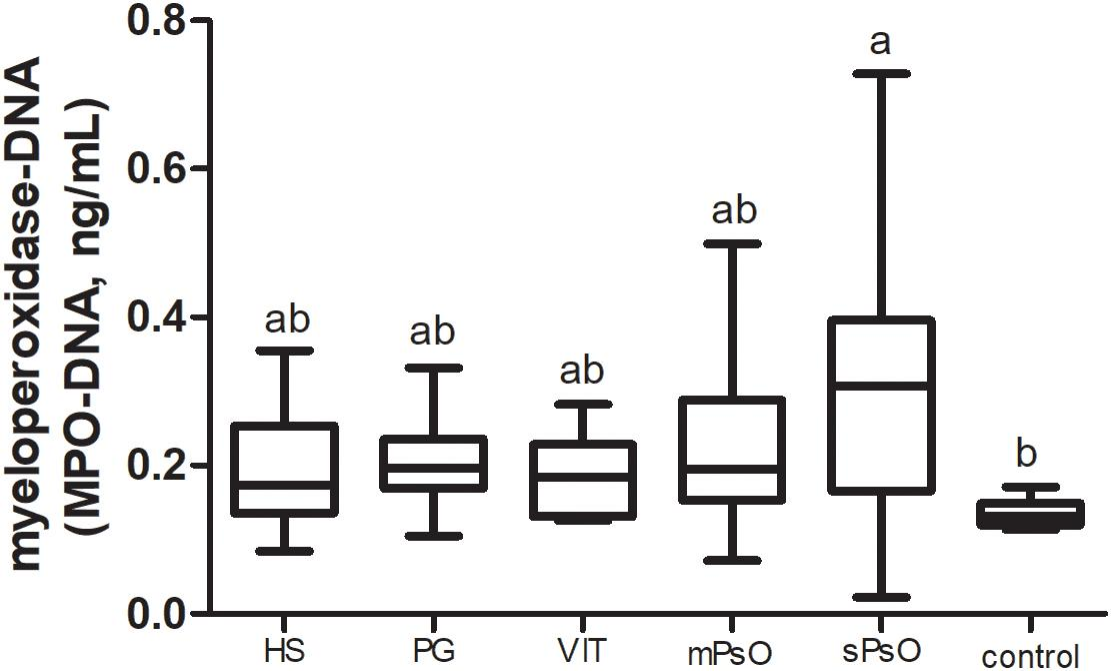
The median (interquartile range, IQR) level of myeloperoxidase–DNA complexes (ng/mL) in the blood serum of patients with Hidradenitis suppurativa (HS, *n* = 13), Pyoderma Gangrenosum (PG, *n* = 11), Vitiligo (VIT, *n* = 13), Mild Psoriasis (mPsO, *n* = 24), Moderate-to-severe Psoriasis (sPsO, *n* = 29), and in the control group (*n* = 30). Differences among groups were assessed using the Kruskal–Wallis test followed by *post hoc* multiple comparisons [H(5) = 14.802, *p* = 0.011]. Different letters above the boxplots indicate statistically significant pairwise differences (*p* < 0.05), whereas groups sharing at least one letter are not significantly different. A significant difference was observed only between sPsO and healthy controls (*p* = 0.004), with higher MPO–DNA levels in sPsO. No other pairwise comparisons were statistically significant.

**Figure 2:**
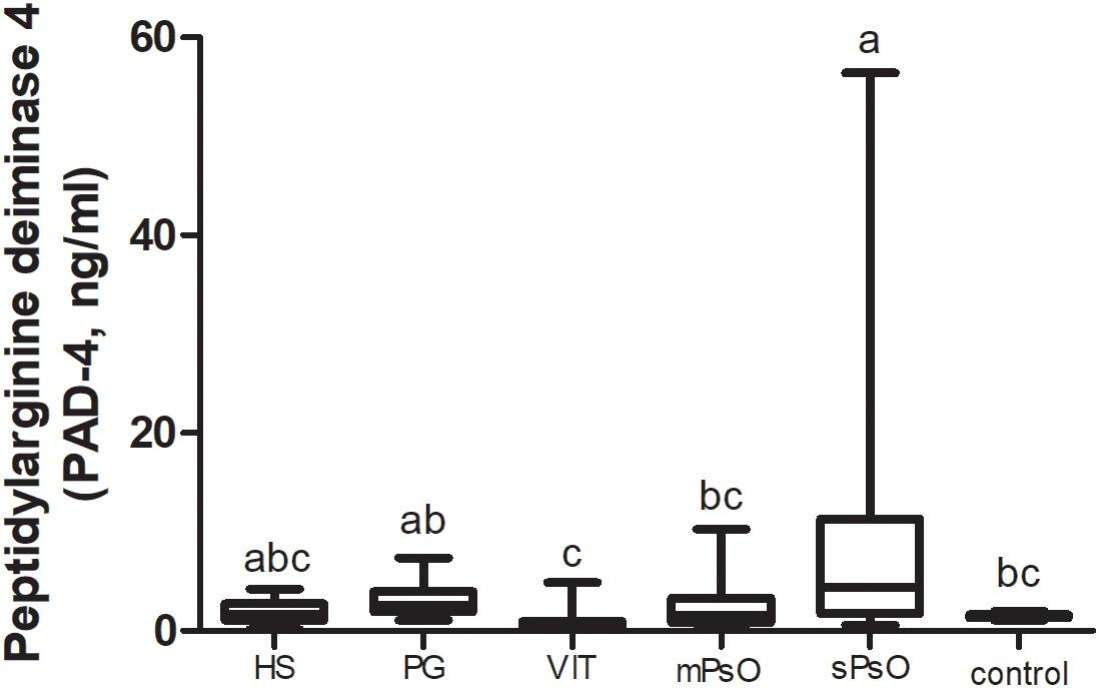
The median (interquartile range, IQR) level of peptidylarginine deiminase 4 (PAD-4, ng/mL) in the blood serum of the patients with hidradenitis suppurativa (HS, *n* = 13), pyoderma gangrenosum (PG, *n* = 11), vitiligo (VIT, *n* = 13), mild psoriasis (mPsO, *n* = 24) and moderate to severe psoriasis (sPsO, *n* = 29) and in the control group (*n* = 30). Differences among groups were assessed using the Kruskal–Wallis test followed by *post hoc* multiple comparisons [H(5) = 35.664, *p* < 0.001]. Different letters above the boxplots indicate statistically significant pairwise differences (*p* < 0.05), whereas groups sharing at least one letter are not significantly different. Significant differences were observed between PG and VIT (*p* = 0.001), sPsO and VIT (*p* < 0.001), sPsO and mPsO (*p* = 0.020), and sPsO and healthy controls (*p* = 0.012). No other pairwise comparisons were statistically significant.

**Figure 3:**
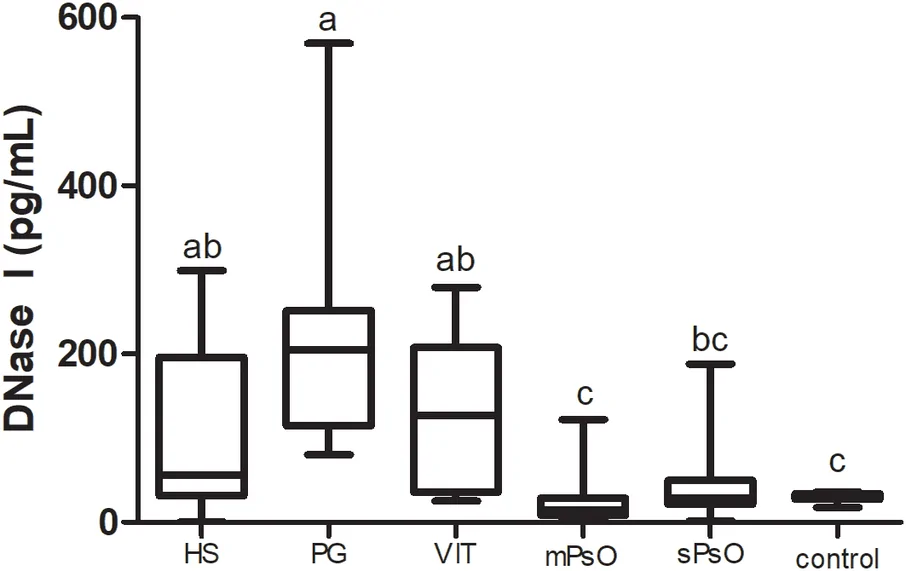
The median (interquartile range, IQR) level of DNase I (pg/mL) in the blood serum of the patients with hidradenitis suppurativa (HS, *n* = 13), pyoderma gangrenosum (PG, *n* = 11), vitiligo (VIT, *n* = 13), mild psoriasis (mPsO, *n* = 24) and moderate to severe psoriasis (sPsO, *n* = 29) and in the control group (*n* = 30). Differences among groups were assessed using the Kruskal–Wallis test followed by *post hoc* multiple comparisons. Different letters indicate statistically significant differences between groups (*p* < 0.05), whereas groups sharing at least one letter did not differ significantly. HS differed significantly from mPsO (*p* < 0.001) and healthy controls (*p* = 0.043); PG differed significantly from mPsO (*p* < 0.001), sPsO (*p* < 0.001), and healthy controls (*p* < 0.001); and VIT differed significantly from mPsO (*p* < 0.001) and healthy controls (*p* = 0.004).

**Table 1:** Mean ± SD of circulating NETosis-related markers (MPO–DNA complexes, PAD-4, DNase I) across study groups.

Type of disease	MPO-DNA (ng/mL)	PAD-4 (ng/mL)	DNase I (pg/mL)
Hidradenitis suppurativa	0.16 ± 0.06	1.91 ± 1.24	107.58 ± 102.64
Pyoderma gangrenosum	0.20 ± 0.06	3.12 ± 2.04	207.90 ± 136.43
Vitiligo	0.19 ± 0.05	0.87 ± 1.27	121.12 ± 86.88
Mild psoriasis	0.23 ± 0.12	2.41 ± 2.39	27.22 ± 32.82
Moderate to severe psoriasis	0.31 ± 0.17	15.02 ± 36.83	41.45 ± 37.88
Healthy control	0.19 ± 0.13	1.64 ± 0.53	26.02 ± 7.53

The Kruskal–Wallis test revealed a significant overall difference in MPO–DNA levels among the study groups [H(5) = 14.802, *p* = 0.011, *ε*² = 0.086, 95% CI: 0.009–0.267]. *Post hoc* multiple comparisons of mean ranks showed that MPO–DNA levels were significantly higher in patients with moderate-to-severe psoriasis (0.31 ± 0.17 ng/mL) than in healthy controls (0.19 ± 0.13 ng/mL; Bonferroni-adjusted *p* = 0.004), whereas no other pairwise comparisons reached statistical significance.

PAD-4 levels differed significantly among the study groups [Kruskal–Wallis H(5) = 35.664, *p* < 0.001, *ε*² = 0.269, 95% CI: 0.142–0.462]. Bonferroni-adjusted *post hoc* multiple comparisons of mean ranks showed significantly higher PAD-4 levels in PG than in VIT (*p* = 0.001). Moderate-to-severe psoriasis (15.02 ± 36.83 ng/mL) showed significantly higher PAD-4 levels than VIT (0.87 ± 1.27 ng/mL, *p* < 0.001), mild psoriasis (2.41 ± 2.39 ng/mL, *p* = 0.020), and healthy controls (1.64 ± 0.53 ng/mL, *p* = 0.012). No other pairwise comparisons reached statistical significance.

DNase I levels differed significantly among the study groups [Kruskal–Wallis H(5) = 52.214, *p* < 0.001, *ε*² = 0.414, 95% CI: 0.311–0.574]. Bonferroni-adjusted *post hoc* multiple comparisons of mean ranks showed significantly higher DNase I levels in HS (107.58 ± 102.64 pg/mL) than in mild psoriasis (27.22 ± 32.82 pg/mL, *p* < 0.001) and healthy controls (26.02 ± 7.53 pg/mL, *p* = 0.043). PG showed significantly higher DNase I levels (207.90 ± 136.43 pg/mL) than mild psoriasis (27.22 ± 32.82 pg/mL, *p* < 0.001), moderate-to-severe psoriasis (41.45 ± 37.88 pg/mL, *p* < 0.001), and healthy controls (26.02 ± 7.53 pg/mL, *p* < 0.001). Similarly, DNase I levels in VIT (121.12 ± 86.88 pg/mL) were significantly higher than those in mild psoriasis (27.22 ± 32.82 pg/mL, *p* < 0.001) and healthy controls (26.02 ± 7.53 pg/mL, *p* = 0.004). No other pairwise differences reached statistical significance.

An exploratory NET balance index (eNET) was additionally evaluated to assess the relative relationship between circulating NET-associated formation and degradation markers. The index showed disease-dependent variation; however, given its exploratory and non-validated nature, these findings should be interpreted cautiously. Detailed eNET results are provided in the Supplementary Material 1.

### 2.3 Correlation analyses

In the whole cohort, CRP levels showed a moderate positive correlation with absolute neutrophil counts (Spearman's r = 0.405, *p* < 0.001). Disease-specific correlation analyses were subsequently performed, with the Benjamini–Hochberg procedure applied to control the false discovery rate (FDR) within each disease group. In HS, an inverse correlation between CRP and PAD-4 was observed in the unadjusted analysis (Spearman's r = −0.664, *p* = 0.013), but it did not remain statistically significant after FDR correction (adjusted *p* = 0.373) (Supplementary Material 2). In PG, no significant correlations were observed between CRP, neutrophil counts, and NET-associated parameters (MPO–DNA, DNase I, and PAD-4), either before or after FDR correction (all adjusted *p* > 0.05) (Supplementary Material 3). In VIT, an inverse correlation between CRP and DNase I reached nominal significance (r = −0.559, *p* = 0.047) but did not remain significant after FDR correction (adjusted *p* = 0.377). In contrast, the strong positive correlation between VASI and BSA (r = 0.920, *p* < 0.001) remained significant after correction (adjusted *p* < 0.001). No correlations between NET-associated parameters and clinical disease severity measures remained significant after FDR correction in VIT (Supplementary Material 4). In mPsO, a strong positive correlation between BSA and PASI (r = 0.803, *p* < 0.001) remained significant after FDR correction (adjusted *p* < 0.001), whereas no correlations between NET-associated parameters and inflammatory or clinical measures remained statistically significant after correction (Supplementary Material 5). In sPsO, the positive correlation between BSA and PASI (r = 0.675, *p* < 0.001) remained statistically significant after FDR correction (adjusted *p* = 0.002). Although several nominal associations involving NET-associated parameters were observed in the unadjusted analyses, none remained statistically significant after FDR correction (Supplementary Material 6). In healthy controls, a positive correlation between MPO–DNA and PAD-4 reached nominal significance (r = 0.436, *p* = 0.016), but did not remain statistically significant after FDR correction (adjusted *p* = 0.161). No other correlations between NET-associated parameters and conventional inflammatory parameters were statistically significant after correction (Supplementary Material 7).

## 3 Materials and methods

### 3.1 Study population

The study group included 90 patients (with HS, *n* = 13; PG, *n* = 11; vitiligo, *n* = 13; mild psoriasis, *n* = 24; moderate/severe psoriasis, *n* = 29) treated in the Clinic and at the Department of Dermatology, Sexually Transmitted Diseases and Clinical Immunology in Olsztyn (Poland). Patients with other inflammatory diseases, neoplastic diseases, previous cardiovascular complications, as well as heart, kidney, and liver failure were excluded. The control group consisted of 30 healthy volunteers (12 women and 18 men) with no personal or family history of inflammatory or autoimmune diseases and a mean BMI of 25.87 ± 2.90 kg/m^2^. Controls were recruited independently and were not individually matched to the disease groups. Detailed information on smoking status, comorbidities, and medication use was not systematically collected and therefore could not be incorporated into matching or adjusted analyses. The study was approved by the Bioethical Committee of the Warmia and Mazury University in Olsztyn (Poland) (Resolution 1/2013, 6/2026, 7/2026). Informed consent was obtained from each patient enrolled in the study. Disease activity was assessed at each visit by a dermatologist using the following tools: Hurley Staging and the International Hidradenitis Suppurativa Severity Score System (IHS4-e), as well as the Dermatology Life Quality Index (DLQI) for hidradenitis suppurativa (HS); the Vitiligo Area Scoring Index (VASI) and Body Surface Area (BSA) for vitiligo; and the Psoriasis Area and Severity Index (PASI), BSA, and DLQI for psoriasis (Table 2). Patients were stratified into three groups: mild psoriasis (mPsO): BSA ≤10%, PASI ≤10, and DLQI ≤10, and moderate-to-severe psoriasis (sPsO): (BSA >10% or PASI >10) and DLQI >10 (27). All participants underwent a treatment washout period before blood sampling. A minimum washout period of 4 weeks was required for systemic treatment and 2 weeks for topical treatment. Only patients who were not receiving active systemic or topical treatment at the time of sample collection were included, in order to minimize potential pharmacological effects on circulating NET-associated parameters.

**Table 2:** Demographic, inflammatory, and clinical characteristics of the study groups.

Group	Parameter	Mean ± SD	Median	Min–max
HS (*n* = 13)	Age, years	47.38 ± 9.34	49.00	28–60
	CRP, mg/L	9.45 ± 8.83	7.56	0.6–35.3
	Neutrophils, ×10^3^/µL	6.95 ± 1.80	7.33	4.11–9.42
	Hurley stage	2.69 ± 0.47	3.00	1–3
	IHS4	14.16 ± 8.87	11.00	9–38
	DLQI	17.85 ± 3.03	18.00	14–25
PG (*n* = 11)	Age, years	58.09 ± 19.73	66.00	30–85
	CRP, mg/L	13.94 ± 11.51	10.40	3.7–42.9
	Neutrophils, ×10^3^/µL	5.91 ± 3.48	5.42	2.50–12.95
VIT (*n* = 13)	Age, years	50.00 ± 12.44	48.00	33–72
	CRP, mg/L	1.93 ± 1.05	1.80	0.6–3.8
	Neutrophils, ×10^3^/µL	4.19 ± 1.55	3.98	2.62–8.69
	VASI	2.71 ± 2.74	2.00	0.47–10.35
	BSA, %	3.30 ± 3.14	3.25	0.5–11.5
mPsO (*n* = 24)	Age, years	37.00 ± 11.46	33.50	23–64
	CRP, mg/L	0.70 ± 0.29	0.60	0.6–1.63
	Neutrophils, ×10^3^/µL	3.79 ± 1.22	4.01	1.98–6.30
	PASI	3.66 ± 1.62	3.65	1–7
	BSA, %	3.46 ± 2.89	2.50	0.6–9.25
	DLQI	5.25 ± 3.24	5.50	0–10
sPsO (*n* = 29)	Age, years	49.10 ± 12.76	47.00	24–70
	CRP, mg/L	1.09 ± 0.70	1.00	0.2–3.2
	Neutrophils, ×10^3^/µL	4.61 ± 1.94	4.55	2.13–9.09
	PASI	33.79 ± 19.06	30.00	11–75
	BSA, %	16.90 ± 4.90	18.20	5.4–24.8
	DLQI	20.69 ± 5.58	20.00	11–29
Controls (*n* = 30)	Age, years	39.17 ± 11.02	37.50	21–57
	CRP, mg/L	0.89 ± 0.51	0.60	0.4–2.3
	Neutrophils, ×10^3^/µL	2.51 ± 0.86	2.24	1.66–4.71

Data are presented as mean ± SD, median (range).

HS, hidradenitis suppurativa; PG, pyoderma gangrenosum; VIT, vitiligo; mPsO, mild psoriasis; sPsO, moderate-to-severe psoriasis; CRP, C-reactive protein; IHS4, international hidradenitis suppurativa severity score system; DLQI, dermatology life quality index; VASI, vitiligo area scoring index; BSA, body surface area; PASI, psoriasis area and severity index.

### 3.2 Methods

Participants from all study groups had their blood samples subjected to hematological and biochemical analyses, including levels of neutrophils and C-reactive protein (CRP) (measured by Mindray BC 760CS, Cormay Diagnostics, and Cobas Pure, Roche Diagnostics), which were performed in the Laboratory of the Municipal Polyclinical Hospital in Olsztyn. The DNA-MPO, DNase I and PAD-4 levels in blood serum were assessed by the enzyme-linked immunosorbent assay (ELISA). Serum samples were stored at −80 °C and thawed only once immediately before analysis; repeated freeze–thaw cycles were avoided. Commercially available kits (Enlibio, China and Biorbyt, UK) were used: 0.312–20 ng/mL for MPO-DNA, 78.13–5,000 pg/mL for DNase I, and 0.32–20 ng/mL for PAD-4. The analytical procedures were performed according to the manufacturer's protocol. The assay's validity was confirmed by parallelism between the standard curve and dilutions of randomly selected serum samples. The intra- and inter-assay coefficient of variation for factors was <8% and <12%, respectively. The sensitivity of these assays was defined as the lowest concentration that could be distinguished from zero in the samples, and it was determined to be 0.06 ng/mL (MPO-DNA), 30 pg/mL (DNase I), and 0.121 ng/mL for PAD-4. Absorbance values were measured at 450 nm using Multiskan FC (Thermo Fisher Scientific, Waltham, MA, USA). Detailed technical characteristics of the ELISA assays are provided in Supplementary Material 8. The de-identified raw data supporting the findings of this study are available as Supplementary Material 9 accompanying this article. As an additional exploratory analysis, a NET balance index (eNET) was calculated as (MPO–DNA × PAD-4)/DNase I to provide a hypothesis-generating estimate of the relative balance between circulating NET-associated formation and degradation markers. As this index has not been externally validated, it was analyzed only as an exploratory parameter, with detailed results provided in the Supplementary Material 1.

### 3.3 Statistical analysis

Data distributions were assessed for normality, and non-parametric statistical methods were applied because the analyzed variables were not normally distributed. Differences among study groups were assessed using the Kruskal–Wallis test. When the overall Kruskal–Wallis test was statistically significant, *post hoc* multiple comparisons of mean ranks were performed for all pairs of groups, with Bonferroni-adjusted two-sided *p*-values.

Associations between variables were assessed using Spearman's rank correlation coefficient. To account for multiple testing in the correlation analyses, the Benjamini–Hochberg procedure was applied to control the false discovery rate (FDR), with correction performed separately within each disease-specific correlation matrix. Both unadjusted and FDR-adjusted *p*-values are reported where relevant.

Effect sizes for the overall Kruskal–Wallis analyses were estimated using epsilon-squared (*ε*^2^), calculated as (H − k + 1)/(N − k), where H represents the Kruskal–Wallis test statistic, k the number of groups, and N the total sample size. Ninety-five percent confidence intervals for Spearman's correlation coefficients were estimated using non-parametric bootstrap resampling with 20,000 iterations and the percentile method. Primary statistical analyses were conducted using Statistica software (version 13, StatSoft Inc., Tulsa, OK, USA), whereas bootstrap confidence intervals for *ε*² were calculated using Python (version 3.13.5) with SciPy (version 1.17.0).

No *a priori* sample size or power calculation was performed because the study was exploratory and based on the available cohort rather than designed as a prospectively powered confirmatory study. Statistical significance was defined as *p* < 0.05, including FDR-adjusted *p* < 0.05 for correlation analyses. Results are presented as means ± SD in the tables and as medians with interquartile ranges (IQRs) in the figures. Given the exploratory nature of the study, the observed between-group differences and associations should be interpreted as hypothesis-generating findings requiring independent confirmation rather than as confirmatory evidence or evidence of causal relationships.

## 4 Discussion

Although neutrophil extracellular traps (NETs) are increasingly recognized as components of inflammatory responses, their contribution to chronic inflammatory dermatoses remains incompletely understood. In this exploratory study, we comparatively evaluated circulating NET-associated parameters across inflammatory skin diseases with distinct immunopathological characteristics, including hidradenitis suppurativa (HS), pyoderma gangrenosum (PG), vitiligo (VIT), and psoriasis (PsO). VIT was included as a biologically contrasting chronic inflammatory dermatosis with a distinct immunopathogenic profile rather than as a neutrophilic dermatosis. The aim of this comparative approach was to determine whether circulating NET-associated parameters display heterogeneous patterns across diseases with different immunological backgrounds.

The present analyses revealed significant between-group differences in all three NET-associated parameters, although the patterns differed substantially among individual markers. For MPO–DNA, the only significant pairwise difference was observed between moderate-to-severe psoriasis and healthy controls, with higher concentrations in the former group. DNase I showed a different distribution, with higher concentrations in HS than in mild psoriasis and healthy controls, in PG than in mild psoriasis, moderate-to-severe psoriasis, and healthy controls, and in VIT than in mild psoriasis and healthy controls. PAD-4 concentrations were higher in PG than in VIT, while moderate-to-severe psoriasis showed higher PAD-4 concentrations than VIT, mild psoriasis, and healthy controls. These findings therefore support heterogeneity of circulating NET-associated profiles across the investigated dermatoses rather than a uniform increase in NET-associated markers across inflammatory skin diseases.

Interpretation of these differences requires consideration of what each circulating parameter represents. MPO–DNA complexes provide an indirect measure of circulating NET-associated remnants but do not directly quantify ongoing NET formation within tissues. Similarly, PAD-4 concentrations reflect a circulating NET formation-associated parameter rather than local PAD-4 enzymatic activity. DNase I was measured by ELISA and therefore reflects circulating protein concentration rather than enzymatic activity. Consequently, higher DNase I concentrations cannot be interpreted as evidence of more efficient NET degradation, nor can lower concentrations be assumed to indicate impaired NET clearance. The observed patterns should therefore be regarded as differences in circulating NET-associated parameters rather than direct evidence of altered NET formation or degradation.

HS is characterized by complex dysregulation of innate and adaptive immune pathways, including prominent IL-23/Th17 signaling (28–30). In the present study, MPO–DNA and PAD-4 concentrations in HS did not differ significantly from healthy controls, whereas DNase I concentrations were significantly higher. HS also showed higher DNase I concentrations than mild psoriasis, but not than moderate-to-severe psoriasis. These findings do not support a generalized systemic elevation of all investigated NET-associated markers in HS. Instead, they suggest a distinct circulating profile in which differences in DNase I concentration are more apparent than differences in MPO–DNA or PAD-4. This observation can be considered in the context of previous reports describing alterations in NET-related processes in HS (31–34), although differences in study design, disease severity, biological material, and methods used to assess NET-related processes limit direct comparisons. Importantly, the present results cannot establish whether increased circulating DNase I concentration reflects a compensatory response to NET-associated material, altered regulation of extracellular DNA, or another inflammatory process.

PG showed a similarly distinct rather than uniformly increased NET-associated profile. DNase I concentrations were significantly higher in PG than in mild psoriasis, moderate-to-severe psoriasis, and healthy controls, whereas PAD-4 concentrations were significantly higher than in VIT. In contrast, MPO–DNA concentrations did not differ significantly between PG and healthy controls or the other disease groups. Given the prominent neutrophilic inflammation characteristic of PG, the absence of a corresponding increase in circulating MPO–DNA illustrates that tissue-level neutrophilic activity may not necessarily be reflected by circulating NET-associated remnants. The relatively high DNase I concentrations observed in PG may indicate differences in systemic regulation of extracellular DNA; however, because DNase I activity was not assessed, the biological significance of this finding remains uncertain (16, 35, 36). Tissue-level studies combined with functional degradation assays will be required to determine whether this circulating pattern corresponds to altered local NET turnover in PG.

VIT demonstrated another distinct pattern. PAD-4 concentrations were significantly lower than those observed in PG and moderate-to-severe psoriasis, while DNase I concentrations were significantly higher than in mild psoriasis and healthy controls. However, neither MPO–DNA nor PAD-4 differed significantly between VIT and healthy controls. Thus, rather than indicating a generally “lower NET profile,” the present findings point to a different relative distribution of individual NET-associated parameters in VIT. Given the established importance of oxidative stress in vitiligo, previous studies have suggested that reactive oxygen species may influence neutrophil responses and NET-related pathways (37–39). Nevertheless, oxidative stress and functional NET formation were not directly assessed in the present study, and the observed circulating PAD-4 and DNase I patterns cannot establish a specific oxidative or PAD-4-independent mechanism.

The findings in psoriasis were particularly noteworthy because differences were concentrated predominantly in the moderate-to-severe disease group. MPO–DNA concentrations were significantly higher in moderate-to-severe psoriasis than in healthy controls, while no significant difference was observed between mild psoriasis and controls. Similarly, PAD-4 concentrations were significantly higher in moderate-to-severe psoriasis than in mild psoriasis, VIT, and healthy controls. These results are consistent with previous evidence implicating neutrophil activation and NET-associated processes in psoriatic inflammation (40–44) and suggest that alterations in selected circulating NET-associated parameters may be more evident in patients with a greater inflammatory disease burden. However, the relationship with psoriasis severity requires careful interpretation. Although moderate-to-severe psoriasis differed from mild psoriasis in PAD-4 concentrations and from healthy controls in both PAD-4 and MPO–DNA, within-group correlation analyses did not demonstrate significant associations between these NET-associated parameters and PASI or BSA after correction for multiple comparisons. Thus, the between-group findings should not be interpreted as evidence of a continuous relationship between circulating NET-associated markers and clinical disease severity. The distinction between group-level differences and individual-level correlations is important: patients classified as having moderate-to-severe psoriasis may differ from those with mild disease in multiple biological and clinical characteristics that are not captured by PASI alone. Accordingly, the present results suggest a disease-severity-associated group phenotype rather than establishing MPO–DNA or PAD-4 as quantitative markers of psoriasis severity. DNase I further illustrates the complexity of the psoriasis findings. Unlike MPO–DNA and PAD-4, DNase I did not show a progressive pattern corresponding to psoriasis severity. Mild psoriasis had significantly lower DNase I concentrations than HS, PG, and VIT, whereas moderate-to-severe psoriasis differed significantly only from PG. Moreover, DNase I concentrations did not differ significantly between either psoriasis subgroup and healthy controls. Taken together, these findings do not support a simple model in which increasing psoriasis severity is accompanied by increased NET formation and reduced NET degradation. Instead, the different distributions of MPO–DNA, PAD-4, and DNase I suggest that individual components of NET-associated biology may vary independently and that their circulating concentrations cannot be interpreted as a direct formation–degradation continuum.

Correlation analyses further supported a cautious interpretation of the circulating NET-associated parameters. After Benjamini–Hochberg correction for multiple testing, no significant correlations between NET-associated parameters and conventional inflammatory or clinical disease severity measures remained within the disease groups. Although several nominal associations were observed in the unadjusted analyses, including associations involving MPO–DNA and PAD-4 in moderate-to-severe psoriasis, none remained statistically significant after FDR correction. Similarly, in healthy controls, the nominal positive correlation between MPO–DNA and PAD-4 did not remain significant after FDR correction. In contrast, correlations between established clinical measures, including VASI and BSA in VIT and BSA and PASI in both psoriasis groups, remained significant after correction. Overall, the limited correspondence between NET-associated parameters, CRP, neutrophil counts, and clinical severity measures suggests that these circulating parameters do not simply mirror conventional systemic inflammation; however, the present study cannot establish their independence from systemic inflammatory activity.

Collectively, our findings demonstrate heterogeneous patterns of circulating NET-associated parameters across chronic inflammatory dermatoses. Rather than identifying a single disease-specific “NET signature,” the results indicate that MPO–DNA, PAD-4, and DNase I show partly independent distributions across diseases. Moderate-to-severe psoriasis was distinguished by higher MPO–DNA and PAD-4 concentrations in selected pairwise comparisons, whereas HS, PG, and VIT were characterized predominantly by differences in DNase I concentration. These observations may provide complementary information for investigating inflammatory heterogeneity across dermatoses, but they do not establish the clinical utility of these parameters for disease discrimination, activity assessment, or patient stratification.

As an additional hypothesis-generating approach, we explored a composite eNET index incorporating MPO–DNA, PAD-4, and DNase I. Descriptive eNET values varied across disease groups, with the highest value observed in moderate-to-severe psoriasis. However, because this index has not been externally validated and combines circulating surrogate parameters with different biological meanings, it should not be interpreted as a quantitative measure of NET formation, degradation, clearance, or turnover. The eNET analysis was therefore restricted to descriptive purposes, with detailed results provided in Supplementary Material 1.

Several limitations should be considered when interpreting these findings. First, the sample sizes of the individual disease groups were relatively small, limiting statistical power and increasing uncertainty around subgroup estimates. The study was exploratory and hypothesis-generating; although *post hoc* pairwise comparisons following significant Kruskal–Wallis tests incorporated adjustment for multiple comparisons and correlation analyses were subjected to Benjamini–Hochberg FDR correction, the number of analyses and small subgroup sizes still warrant cautious interpretation of individual findings. Second, the cross-sectional design precludes assessment of temporal relationships or causal mechanisms. Third, all NET-associated parameters were measured in the circulation and therefore cannot directly reflect NET formation, persistence, or degradation within lesional skin. MPO–DNA complexes represent circulating NET-associated remnants rather than a direct measure of ongoing NET formation, PAD-4 concentration does not directly measure PAD-4 enzymatic activity, and DNase I concentration does not represent functional DNase I activity and may be influenced by endogenous regulatory factors. Fourth, the study did not include additional orthogonal NET-associated markers, such as cell-free DNA, citrullinated histone H3, or neutrophil elastase, nor did it include functional NET formation or degradation assays. In psoriasis specifically, LL-37/cathelicidin–DNA complexes, which may provide greater mechanistic insight into the interaction between neutrophil-derived DNA and psoriatic immune pathways, were not assessed. Fifth, the healthy control group was not formally matched to the disease groups for age, sex, BMI, smoking status, comorbidities, or medication use. Moreover, detailed information on smoking status, comorbidities, and medication use was not systematically collected in controls; therefore, residual confounding related to these factors cannot be excluded. Future studies incorporating larger and appropriately matched cohorts, longitudinal sampling, tissue-level analyses, orthogonal NET-associated markers, and functional assays are required to validate and mechanistically interpret the circulating NET-associated patterns observed in the present study.

## Data Availability

The original contributions presented in the study are included in the article/Supplementary Material, further inquiries can be directed to the corresponding author.
