# Supplementary material for: Circulating NET-associated parameters across chronic inflammatory skin diseases: a comparative study
Source: Front Med (Lausanne). 2026 Oct 23;13:1918335. doi: 10.3389/fmed.2026.1918335 (PMC13739568; doi:10.3389/fmed.2026.1918335)
Supplement: Supplementary file 1 [file Table1.docx]

Supplementary material 1

An exploratory composite index (eNET) was calculated as (MPO–DNA × PAD-4)/DNase I to provide a descriptive summary of the relative distribution of the three circulating NET-associated parameters across disease groups. This in-house index has not been externally validated and should not be interpreted as a direct or quantitative measure of NET formation, degradation, clearance, or turnover.

The descriptive eNET values were as follows: moderate-to-severe psoriasis (0.10), mild psoriasis (0.02), hidradenitis suppurativa (0.003), pyoderma gangrenosum (0.003), and vitiligo (0.001). Given the exploratory and non-validated nature of this index, these values were used solely for descriptive, hypothesis-generating comparisons and were not subjected to inferential statistical testing.

Table 1. Mean ± SD of circulating NETosis-related markers (MPO–DNA complexes, PAD-4, DNase I) across study groups. The table also includes the eNET index as an exploratory composite parameter.

|  | MPO-DNA (ng/mL) | PAD-4 (ng/mL) | DNAse I (pg/mL) | eNET index |
| --- | --- | --- | --- | --- |
| Hirradenitis suppurativa | 0.16±0.06 | 1.91±1.24 | 107.58±102.64 | 0.003 |
| Pyoderma gangrenosum | 0.20±0.06 | 3.12±2.04 | 207.90±136.43 | 0.003 |
| Vitiligo | 0.19±0.05 | 0.87±1.27 | 121.12±86.88 | 0.001 |
| Mild psoriasis | 0.23±0.12 | 2.41±2.39 | 27.22±32.82 | 0.02 |
| Moderate to severe psoriasis | 0.31±0.17 | 15.02±36.83 | 41.45±37.88 | 0.1 |

Fig.1. Disease-specific NET balance signatures across chronic inflammatory skin diseases. Created in https://BioRender.com
