# Supplementary material for: Circulating NET-associated parameters across chronic inflammatory skin diseases: a comparative study
Source: Front Med (Lausanne). 2026 Oct 23;13:1918335. doi: 10.3389/fmed.2026.1918335 (PMC13739568; doi:10.3389/fmed.2026.1918335)
Supplement: Supplementary file 2 [file Table2.docx]

Supplementary Material 2. Spearman correlation analysis of clinical, inflammatory, and NET-associated parameters in patients with Hidradenitis suppurativa. P-values were adjusted for multiple comparisons using the Benjamini–Hochberg false discovery rate (FDR) procedure. Ninety-five percent confidence intervals (95% CIs) for Spearman’s correlation coefficients were estimated using non-parametric bootstrap resampling with 20,000 iterations and the percentile method. FDR-adjusted p < 0.05 was considered statistically significant.

| **Variable 1** | **Variable 2** | **N** | **Spearman r** | **Raw p** | **FDR-adjusted p** | **95% CI lower** | **95% CI upper** |
| --- | --- | --- | --- | --- | --- | --- | --- |
| mpo | dnase | 13 | 0,420908 | 0,152070 | 0,764586 | -0,291848 | 0,783333 |
| mpo | pad | 13 | -0,077029 | 0,802496 | 0,874888 | -0,659828 | 0,495798 |
| mpo | crp | 13 | -0,360000 | 0,226951 | 0,794327 | -0,864042 | 0,326197 |
| mpo | neutr | 13 | -0,073104 | 0,812396 | 0,874888 | -0,664812 | 0,529185 |
| mpo | hurley | 13 | 0,223024 | 0,463922 | 0,874888 | -0,389946 | 0,717004 |
| mpo | ish4 | 13 | 0,127710 | 0,677572 | 0,874888 | -0,540626 | 0,690195 |
| mpo | dlqi | 13 | -0,121348 | 0,692911 | 0,874888 | -0,694291 | 0,582676 |
| dnase | pad | 13 | -0,098901 | 0,747868 | 0,874888 | -0,585368 | 0,478759 |
| dnase | crp | 13 | -0,261709 | 0,387738 | 0,874888 | -0,715960 | 0,363383 |
| dnase | neutr | 13 | -0,195593 | 0,521916 | 0,874888 | -0,807067 | 0,465919 |
| dnase | hurley | 13 | -0,489979 | 0,089183 | 0,730962 | -0,873252 | 0,246641 |
| dnase | ish4 | 13 | 0,197099 | 0,518652 | 0,874888 | -0,371380 | 0,617543 |
| dnase | dlqi | 13 | 0,387224 | 0,191147 | 0,764586 | -0,268554 | 0,883634 |
| pad | crp | 13 | -0,663914 | 0,013339 | 0,373490 | -0,911401 | -0,146274 |
| pad | neutr | 13 | 0,179064 | 0,558322 | 0,874888 | -0,398490 | 0,720125 |
| pad | hurley | 13 | 0,044544 | 0,885113 | 0,885113 | -0,470029 | 0,588669 |
| pad | ish4 | 13 | 0,168114 | 0,583005 | 0,874888 | -0,470396 | 0,703468 |
| pad | dlqi | 13 | -0,470798 | 0,104423 | 0,730962 | -0,830116 | 0,130863 |
| crp | neutr | 13 | 0,093923 | 0,760217 | 0,874888 | -0,622857 | 0,709402 |
| crp | hurley | 13 | 0,044666 | 0,884798 | 0,885113 | -0,515664 | 0,634150 |
| crp | ish4 | 13 | -0,252866 | 0,404545 | 0,874888 | -0,834814 | 0,508646 |
| crp | dlqi | 13 | 0,100565 | 0,743753 | 0,874888 | -0,658876 | 0,682185 |
| neutr | hurley | 13 | 0,535997 | 0,059012 | 0,730962 | -0,147813 | 0,880663 |
| neutr | ish4 | 13 | 0,296464 | 0,325331 | 0,874888 | -0,340339 | 0,768032 |
| neutr | dlqi | 13 | -0,150847 | 0,622778 | 0,874888 | -0,734762 | 0,385929 |
| hurley | ish4 | 13 | 0,093992 | 0,760044 | 0,874888 | -0,475817 | 0,584716 |
| hurley | dlqi | 13 | -0,406515 | 0,168069 | 0,764586 | -0,861892 | 0,194686 |
| ish4 | dlqi | 13 | -0,183698 | 0,548010 | 0,874888 | -0,745082 | 0,465798 |
