# Supplementary material for: Circulating NET-associated parameters across chronic inflammatory skin diseases: a comparative study
Source: Front Med (Lausanne). 2026 Oct 23;13:1918335. doi: 10.3389/fmed.2026.1918335 (PMC13739568; doi:10.3389/fmed.2026.1918335)
Supplement: Supplementary file 3 [file Table3.docx]

Supplementary Material 3. Spearman correlation analysis of clinical, inflammatory, and NET-associated parameters in patients with Pyoderma Gangrenosum. P-values were adjusted for multiple comparisons using the Benjamini–Hochberg false discovery rate (FDR) procedure. Ninety-five percent confidence intervals (95% CIs) for Spearman’s correlation coefficients were estimated using non-parametric bootstrap resampling with 20,000 iterations and the percentile method. FDR-adjusted p < 0.05 was considered statistically significant.

| **Variable 1** | **Variable 2** | **N** | **Spearman r** | **Raw p** | **FDR-adjusted p** | **95% CI lower** | **95% CI upper** |
| --- | --- | --- | --- | --- | --- | --- | --- |
| mpo | dnase | 11 | -0,245455 | 0,466922 | 0,848949 | -0,880184 | 0,524773 |
| mpo | pad | 11 | 0,136364 | 0,689309 | 0,861636 | -0,481481 | 0,717172 |
| mpo | crp | 11 | 0,022779 | 0,946998 | 0,968186 | -0,679196 | 0,645933 |
| mpo | neutr | 11 | -0,163636 | 0,630685 | 0,861636 | -0,738318 | 0,601897 |
| dnase | pad | 11 | 0,309091 | 0,355028 | 0,848949 | -0,314286 | 0,796117 |
| dnase | crp | 11 | -0,223235 | 0,509370 | 0,848949 | -0,833333 | 0,440248 |
| dnase | neutr | 11 | -0,363636 | 0,271638 | 0,848949 | -0,907407 | 0,383904 |
| pad | crp | 11 | 0,013667 | 0,968186 | 0,968186 | -0,717596 | 0,699029 |
| pad | neutr | 11 | 0,345455 | 0,298089 | 0,848949 | -0,386792 | 0,834101 |
| crp | neutr | 11 | 0,323463 | 0,331890 | 0,848949 | -0,464115 | 0,751126 |
