# Supplementary material for: Circulating NET-associated parameters across chronic inflammatory skin diseases: a comparative study
Source: Front Med (Lausanne). 2026 Oct 23;13:1918335. doi: 10.3389/fmed.2026.1918335 (PMC13739568; doi:10.3389/fmed.2026.1918335)
Supplement: Supplementary file 4 [file Table4.docx]

Supplementary Material 4. Spearman correlation analysis of clinical, inflammatory, and NET-associated parameters in patients with Vitiligo. P-values were adjusted for multiple comparisons using the Benjamini–Hochberg false discovery rate (FDR) procedure. Ninety-five percent confidence intervals (95% CIs) for Spearman’s correlation coefficients were estimated using non-parametric bootstrap resampling with 20,000 iterations and the percentile method. FDR-adjusted p < 0.05 was considered statistically significant.

| **Variable 1** | **Variable 2** | **N** | **Spearman r** | **Raw p** | **FDR-adjusted p** | **95% CI lower** | **95% CI upper** |
| --- | --- | --- | --- | --- | --- | --- | --- |
| mpo | dnase | 13 | -0,346154 | 0,246625 | 0,431594 | -0,787709 | 0,397183 |
| mpo | pad | 13 | -0,472527 | 0,102981 | 0,377117 | -0,915730 | 0,204482 |
| mpo | crp | 13 | 0,396696 | 0,179580 | 0,377117 | -0,195141 | 0,799620 |
| mpo | neutr | 13 | 0,446283 | 0,126351 | 0,377117 | -0,183120 | 0,863576 |
| mpo | VASI | 13 | -0,430946 | 0,141521 | 0,377117 | -0,877097 | 0,204955 |
| mpo | BSA | 13 | -0,192838 | 0,527910 | 0,653690 | -0,789474 | 0,508793 |
| dnase | pad | 13 | 0,401099 | 0,174357 | 0,377117 | -0,348318 | 0,854749 |
| dnase | crp | 13 | -0,559231 | 0,046922 | 0,377117 | -0,837549 | -0,020116 |
| dnase | neutr | 13 | -0,099174 | 0,747193 | 0,747193 | -0,637461 | 0,450081 |
| dnase | VASI | 13 | 0,502770 | 0,079917 | 0,377117 | -0,107528 | 0,876057 |
| dnase | BSA | 13 | 0,515153 | 0,071604 | 0,377117 | -0,117982 | 0,865546 |
| pad | crp | 13 | -0,181819 | 0,552182 | 0,653690 | -0,697280 | 0,418677 |
| pad | neutr | 13 | -0,154271 | 0,614813 | 0,679531 | -0,707737 | 0,472825 |
| pad | VASI | 13 | 0,314922 | 0,294625 | 0,441938 | -0,401153 | 0,805014 |
| pad | BSA | 13 | 0,236915 | 0,435782 | 0,610095 | -0,505000 | 0,798319 |
| crp | neutr | 13 | 0,425414 | 0,147273 | 0,377117 | -0,194105 | 0,801666 |
| crp | VASI | 13 | -0,315791 | 0,293223 | 0,441938 | -0,859333 | 0,324675 |
| crp | BSA | 13 | -0,364641 | 0,220578 | 0,421103 | -0,903473 | 0,321639 |
| neutr | VASI | 13 | -0,099723 | 0,745834 | 0,747193 | -0,599738 | 0,409121 |
| neutr | BSA | 13 | -0,178177 | 0,560306 | 0,653690 | -0,640044 | 0,345863 |
| VASI | BSA | 13 | 0,919671 | 0,000009 | 0,000181 | 0,640549 | 0,994135 |
