# Supplementary material for: Circulating NET-associated parameters across chronic inflammatory skin diseases: a comparative study
Source: Front Med (Lausanne). 2026 Oct 23;13:1918335. doi: 10.3389/fmed.2026.1918335 (PMC13739568; doi:10.3389/fmed.2026.1918335)
Supplement: Supplementary file 5 [file Table5.docx]

Supplementary Material 5. Spearman correlation analysis of clinical, inflammatory, and NET-associated parameters in patients with mild psoriasis. P-values were adjusted for multiple comparisons using the Benjamini–Hochberg false discovery rate (FDR) procedure. Ninety-five percent confidence intervals (95% CIs) for Spearman’s correlation coefficients were estimated using non-parametric bootstrap resampling with 20,000 iterations and the percentile method. FDR-adjusted p < 0.05 was considered statistically significant.

| **Variable 1** | **Variable 2** | **N** | **Spearman r** | **Raw p** | **FDR-adjusted p** | **95% CI lower** | **95% CI upper** |
| --- | --- | --- | --- | --- | --- | --- | --- |
| mpo | dnase | 24 | -0,202958 | 0,341523 | 0,878597 | -0,619270 | 0,287027 |
| mpo | pad | 24 | 0,163874 | 0,444181 | 0,878597 | -0,289716 | 0,569872 |
| mpo | crp | 24 | -0,104330 | 0,627569 | 0,878597 | -0,503917 | 0,335850 |
| mpo | neutr | 24 | 0,030686 | 0,886815 | 0,960922 | -0,422348 | 0,467659 |
| mpo | BSA | 24 | -0,029630 | 0,890686 | 0,960922 | -0,392693 | 0,335322 |
| mpo | PASI | 24 | 0,131997 | 0,538666 | 0,878597 | -0,294606 | 0,506358 |
| mpo | DLQI | 24 | 0,302213 | 0,151197 | 0,604789 | -0,145476 | 0,691778 |
| dnase | pad | 24 | -0,006963 | 0,974242 | 0,995612 | -0,363719 | 0,352742 |
| dnase | crp | 24 | -0,134651 | 0,530461 | 0,878597 | -0,520955 | 0,268286 |
| dnase | neutr | 24 | 0,152742 | 0,476137 | 0,878597 | -0,299714 | 0,534371 |
| dnase | BSA | 24 | -0,180571 | 0,398467 | 0,878597 | -0,543653 | 0,258282 |
| dnase | PASI | 24 | -0,335802 | 0,108659 | 0,507077 | -0,659968 | 0,079198 |
| dnase | DLQI | 24 | -0,106136 | 0,621589 | 0,878597 | -0,491502 | 0,312830 |
| pad | crp | 24 | -0,367367 | 0,077396 | 0,507077 | -0,697940 | 0,035387 |
| pad | neutr | 24 | -0,070309 | 0,744077 | 0,947007 | -0,428699 | 0,341025 |
| pad | BSA | 24 | -0,120288 | 0,575569 | 0,878597 | -0,520843 | 0,308312 |
| pad | PASI | 24 | 0,029194 | 0,892285 | 0,960922 | -0,439687 | 0,494200 |
| pad | DLQI | 24 | 0,156526 | 0,465145 | 0,878597 | -0,308344 | 0,581956 |
| crp | neutr | 24 | 0,001186 | 0,995612 | 0,995612 | -0,440882 | 0,431128 |
| crp | BSA | 24 | 0,338565 | 0,105601 | 0,507077 | -0,074928 | 0,678375 |
| crp | PASI | 24 | 0,198917 | 0,351426 | 0,878597 | -0,220268 | 0,568352 |
| crp | DLQI | 24 | -0,042395 | 0,844067 | 0,960922 | -0,482052 | 0,377387 |
| neutr | BSA | 24 | -0,184572 | 0,387919 | 0,878597 | -0,614240 | 0,289883 |
| neutr | PASI | 24 | -0,089760 | 0,676603 | 0,902137 | -0,516045 | 0,345694 |
| neutr | DLQI | 24 | 0,347786 | 0,095854 | 0,507077 | -0,058602 | 0,664843 |
| BSA | PASI | 24 | 0,803053 | 0,000002 | 0,000065 | 0,541124 | 0,929505 |
| BSA | DLQI | 24 | 0,188828 | 0,376876 | 0,878597 | -0,246609 | 0,558155 |
| PASI | DLQI | 24 | 0,348749 | 0,094876 | 0,507077 | -0,100539 | 0,660194 |
