# Supplementary material for: Circulating NET-associated parameters across chronic inflammatory skin diseases: a comparative study
Source: Front Med (Lausanne). 2026 Oct 23;13:1918335. doi: 10.3389/fmed.2026.1918335 (PMC13739568; doi:10.3389/fmed.2026.1918335)
Supplement: Supplementary file 6 [file Table6.docx]

Supplementary Material 6. Spearman correlation analysis of clinical, inflammatory, and NET-associated parameters in patients with moderate-to-severe psoriasis. P-values were adjusted for multiple comparisons using the Benjamini–Hochberg false discovery rate (FDR) procedure. Ninety-five percent confidence intervals (95% CIs) for Spearman’s correlation coefficients were estimated using non-parametric bootstrap resampling with 20,000 iterations and the percentile method. FDR-adjusted p < 0.05 was considered statistically significant.

| **Variable 1** | **Variable 2** | **N** | **Spearman r** | **Raw p** | **FDR-adjusted p** | **95% CI lower** | **95% CI upper** |
| --- | --- | --- | --- | --- | --- | --- | --- |
| mpo | dnase | 29 | 0,009858 | 0,959521 | 0,994373 | -0,422840 | 0,419571 |
| mpo | pad | 29 | 0,178972 | 0,352926 | 0,858263 | -0,228755 | 0,548356 |
| mpo | crp | 29 | 0,430715 | 0,019675 | 0,183632 | 0,092500 | 0,693552 |
| mpo | neutr | 29 | 0,401553 | 0,030841 | 0,215890 | 0,031427 | 0,684966 |
| mpo | BSA | 29 | 0,076164 | 0,694554 | 0,866467 | -0,351416 | 0,486583 |
| mpo | PASI | 29 | 0,181819 | 0,345193 | 0,858263 | -0,222041 | 0,521992 |
| mpo | DLQI | 29 | -0,173589 | 0,367827 | 0,858263 | -0,558824 | 0,232380 |
| dnase | pad | 29 | -0,104264 | 0,590402 | 0,866467 | -0,444173 | 0,280651 |
| dnase | crp | 29 | 0,112265 | 0,562043 | 0,866467 | -0,308409 | 0,509766 |
| dnase | neutr | 29 | -0,210915 | 0,272089 | 0,846498 | -0,594033 | 0,213373 |
| dnase | BSA | 29 | -0,226611 | 0,237161 | 0,830062 | -0,568146 | 0,161223 |
| dnase | PASI | 29 | 0,070273 | 0,717177 | 0,866467 | -0,298628 | 0,430673 |
| dnase | DLQI | 29 | -0,227987 | 0,234249 | 0,830062 | -0,569880 | 0,164235 |
| pad | crp | 29 | 0,111416 | 0,565021 | 0,866467 | -0,335673 | 0,516033 |
| pad | neutr | 29 | 0,482810 | 0,007982 | 0,111753 | 0,177274 | 0,697873 |
| pad | BSA | 29 | 0,063704 | 0,742686 | 0,866467 | -0,311302 | 0,428818 |
| pad | PASI | 29 | -0,019024 | 0,921972 | 0,992893 | -0,431660 | 0,381899 |
| pad | DLQI | 29 | 0,124025 | 0,521522 | 0,866467 | -0,254890 | 0,469672 |
| crp | neutr | 29 | 0,242773 | 0,204459 | 0,830062 | -0,129992 | 0,564266 |
| crp | BSA | 29 | -0,091020 | 0,638658 | 0,866467 | -0,496862 | 0,322506 |
| crp | PASI | 29 | -0,250524 | 0,189929 | 0,830062 | -0,610104 | 0,160964 |
| crp | DLQI | 29 | -0,074333 | 0,701557 | 0,866467 | -0,451092 | 0,303564 |
| neutr | BSA | 29 | 0,150932 | 0,434488 | 0,866467 | -0,231684 | 0,490519 |
| neutr | PASI | 29 | 0,088047 | 0,649704 | 0,866467 | -0,300238 | 0,457272 |
| neutr | DLQI | 29 | -0,055406 | 0,775289 | 0,868324 | -0,490143 | 0,412698 |
| BSA | PASI | 29 | 0,674997 | 0,000059 | 0,001653 | 0,349824 | 0,869988 |
| BSA | DLQI | 29 | 0,119604 | 0,536587 | 0,866467 | -0,279658 | 0,485827 |
| PASI | DLQI | 29 | 0,001370 | 0,994373 | 0,994373 | -0,410704 | 0,394136 |
