# Supplementary material for: Circulating NET-associated parameters across chronic inflammatory skin diseases: a comparative study
Source: Front Med (Lausanne). 2026 Oct 23;13:1918335. doi: 10.3389/fmed.2026.1918335 (PMC13739568; doi:10.3389/fmed.2026.1918335)
Supplement: Supplementary file 7 [file Table7.docx]

Supplementary Material 7. Spearman correlation analysis of clinical, inflammatory, and NET-associated parameters in control group. P-values were adjusted for multiple comparisons using the Benjamini–Hochberg false discovery rate (FDR) procedure. Ninety-five percent confidence intervals (95% CIs) for Spearman’s correlation coefficients were estimated using non-parametric bootstrap resampling with 20,000 iterations and the percentile method. FDR-adjusted p < 0.05 was considered statistically significant.

| **Variable 1** | **Variable 2** | **N** | **Spearman r** | **Raw p** | **FDR-adjusted p** | **95% CI lower** | **95% CI upper** |
| --- | --- | --- | --- | --- | --- | --- | --- |
| mpo | dnase | 30 | 0,267766 | 0,152553 | 0,508510 | -0,162176 | 0,598906 |
| mpo | pad | 30 | 0,435712 | 0,016096 | 0,160959 | 0,033753 | 0,730734 |
| mpo | crp | 30 | 0,042990 | 0,821541 | 0,941356 | -0,339103 | 0,436817 |
| mpo | neutr | 30 | 0,295761 | 0,112547 | 0,508510 | -0,084008 | 0,639013 |
| dnase | pad | 30 | 0,178241 | 0,346009 | 0,839700 | -0,226264 | 0,546481 |
| dnase | crp | 30 | -0,014027 | 0,941356 | 0,941356 | -0,400169 | 0,372625 |
| dnase | neutr | 30 | 0,035539 | 0,852099 | 0,941356 | -0,344097 | 0,417776 |
| pad | crp | 30 | -0,016557 | 0,930801 | 0,941356 | -0,406850 | 0,358766 |
| pad | neutr | 30 | 0,063666 | 0,738202 | 0,941356 | -0,346650 | 0,463747 |
| crp | neutr | 30 | 0,152907 | 0,419850 | 0,839700 | -0,258747 | 0,524076 |
