# Supplementary material for: Circulating NET-associated parameters across chronic inflammatory skin diseases: a comparative study
Source: Front Med (Lausanne). 2026 Oct 23;13:1918335. doi: 10.3389/fmed.2026.1918335 (PMC13739568; doi:10.3389/fmed.2026.1918335)
Supplement: Supplementary file 8 [file Table8.docx]

Supplementary Material 8. Technical characteristics of ELISA assays used for the assessment of NET-associated markers

| Assay characteristic | MPO–DNA (ng/mL) | PAD-4 (ng/mL) | DNase I (pg/mL) |
| --- | --- | --- | --- |
| Manufacturer | Enlibio, China | Biorbyt, UK | Biorbyt, UK |
| Catalog No. | EIA09433h | orb777768 | Orb775655 |
| Sample type | Serum | Serum | Serum |
| Sample dilution | None | None | None |
| Standard range | 0.32–20 ng/mL | 0.32–20 ng/mL | 78.13–5000 pg/mL |
| Sensitivity | 0.06 ng/mL | 0.121 ng/mL | 30 pg/mL |
| Storage conditions | −80°C | −80°C | −80°C |
| Freeze–thaw cycles before analysis | once | once | once |
| Intra-assay CV | <8% | <8% | <8% |
| Inter-assay CV | <12% | <10% | <10% |
| Dilutional linearity/parallelism | Confirmed | Confirmed | Confirmed |
